# Epigenetically regulated gene expression profiles recognized three molecular classifications with prognostic and therapeutic implications in bladder cancer

**DOI:** 10.1002/ctm2.1145

**Published:** 2023-03-02

**Authors:** Hui Xu, Zaoqu Liu, Siyuan Weng, Yuqing Ren, Jianzhuang Ren, Xinwei Han

**Affiliations:** ^1^ Department of Interventional Radiology The First Affiliated Hospital of Zhengzhou University Zhengzhou China; ^2^ Interventional Institute of Zhengzhou University Zhengzhou China; ^3^ Henan Provincial Health Commission Interventional Treatment and Clinical Research Center of Henan Province Zhengzhou China; ^4^ Department of Respiratory and Critical Care Medicine The First Affiliated Hospital of Zhengzhou University Zhengzhou China

Dear Editor

Transcriptome dysregulation by epigenetics plays a significant role in the heterogeneous characteristic of bladder cancer.^1^, ^2^ However, the epigenetic mechanisms underlying BLCA heterogeneity are unclear and stable epigenetic molecular subtypes are still lacking.

The flow chart of our work is displayed in Figure S1. For a detailed description of methods and materials, please refer to Supporting Information. In our research, only 52 genes overlapped in the miRNA‐correlated (MIRcor) and methylation‐correlated (METcor) genes, revealing that miRNAs and DNA methylation were unlikely to regulate the same genes coordinately (Figure S2A). Besides, DNA METcor genes are often located in CpG islands (Figure S2B) and the methylation frequency of the 1stExon is much higher (Figure S2C), indicating that DNA methylation at CpG islands and 1stExon regions may play a fundamental role in mRNA expression regulation. Results of pathway analysis reveal distinct functional enrichment of MIRcor genes and METcor genes, suggesting that METcor and MIRcor genes tend to exhibit various biological functions in the regulation of downstream genes (Figure S2D,E). Besides, the aberrant frequencies of MIRcor genes and METcor genes were significantly correlated (Figure S2F,G).

Considering significant associations of MIRcor and METcor genes, the integrated cluster analysis base on the union of METcor and MIRcor gene expression profiles was performed to recognize three bladder cancer (BLCA) subtypes (Figure 1A). Kaplan‐Meier analysis indicated a remarked distinct in overall survival among the three classifications, with C2 possessing the best prognosis and C3 displaying the worst prognosis (Figure 1B). To verify this, the NTP algorithm was employed to forecast the subtype of BLCA patients in four independent datasets.^3^ SubMap analysis suggested that all these subtypes in four gene expression omnibus (GEO) datasets were significantly related to the corresponding classifications of TCGA‐BLCA (Figure 1G) and the survival differences among three BLCA classifications were also validated across four GEO cohorts (Figure 1C–F). The differences in the miRNA and methylation profiles of the three subtypes were further explored, and the results showed that the three subtypes differed from each other in the regulation of gene expression by epigenetic aberrations (Figure S3A–C).

**FIGURE 1:**
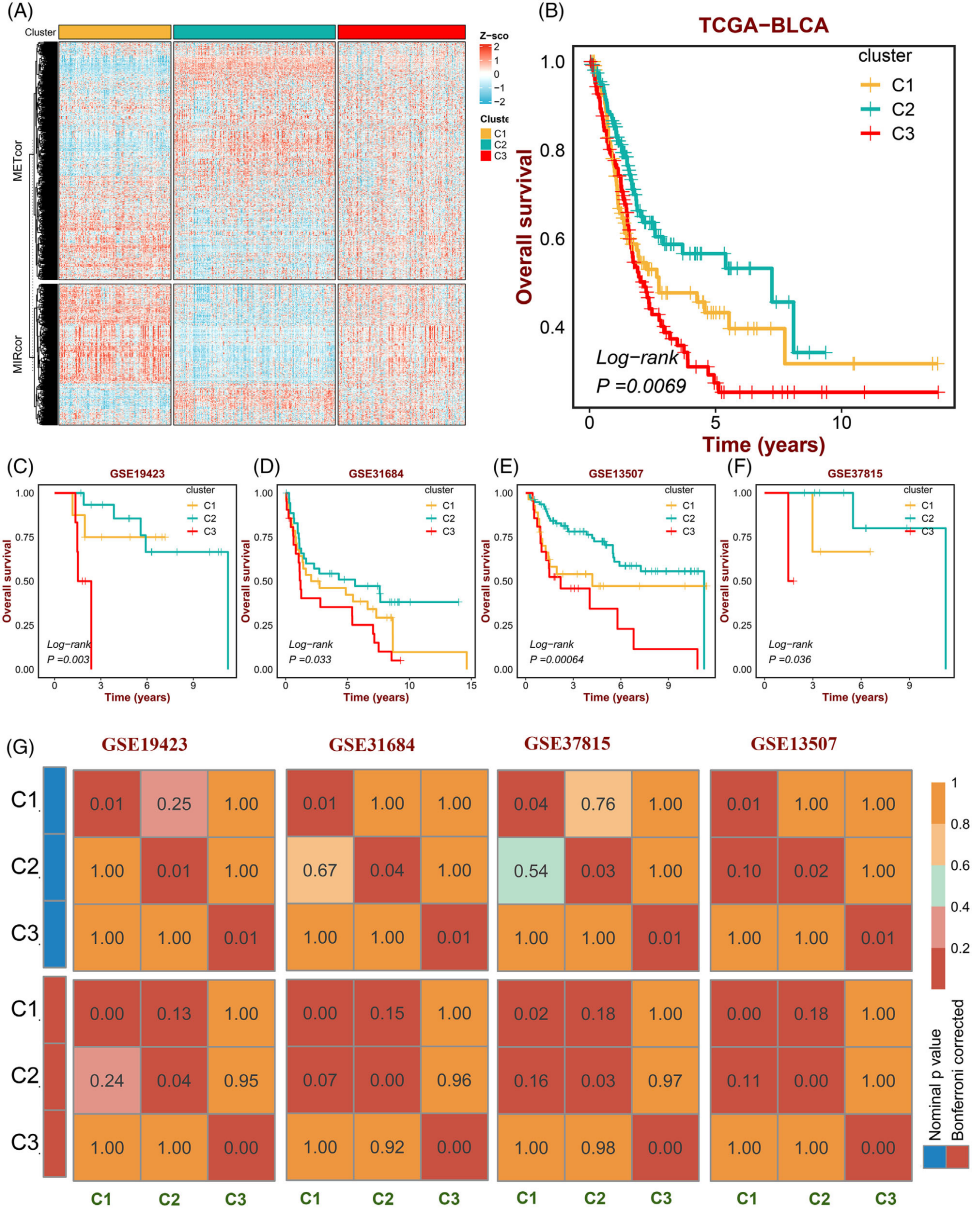
Identification and validation of BLCA subtypes. (A) Heatmaps show gene expression patterns of the BLCA subtypes identified by integrative clustering analysis for methylation‐correlated (METcor) and miRNA‐correlated (MIRcor) data. (B) Kaplan‐Meier survival curves for the BLCA subtypes in the training dataset. (C‐F) Kaplan‐Meier survival curves in the validation dataset (GSE19423, GSEGSE31684, GSE13507 and GSE37815). (G) SubMap analysis shows the similarity of gene expression profiles (GEPs) between the BLCA subtypes of TCGA and GEO datasets

To explore the underlying functional mechanisms of different subtypes, we identified signature genes for each subtype, pathway enrichment analysis revealed significant functional differences among the three molecular subtypes (Figure S4A–D). Specifically, C1 exhibited significant enrichment of immune‐related pathways, C2 mainly displayed the enrichment of metabolic‐related pathways, and C3 mainly enriched in Wnt signalling‐related pathways. The above results were further validated in gene ontology (GO) and Kyoto Encyclopedia of Genes and Gnomes (KEGG) enrichment analyses performed by the Gene Set Enrichment Analysis algorithm (Figure S5A–F).

Further, we compared the clinical features of BLCA classifications in the TCGA‐BLCA and GSE13507 cohorts. C1 was associated with higher grade and non‐papillary tumours; C2 was related to a low‐level stage and papillary tumours; whereas C3 was linked with advanced clinical stage and T stage, respectively (Figure S4E). The same result was also observed in the GSE13507 validation cohort (Figure S4F). Besides, dramatic correlations were obtained between our classification and published subtypes (Figure S4E,F), which were consistent with the unique aggressive properties in our classifications.^4^

To investigate the association between our subtypes and immunity, the immune landscape of three subtypes was explored based on immune cell infiltration and immune checkpoints. As we expect, the C1 subtype exhibited significantly higher immune cell infiltration compared to the other subtypes (Figure 2A,B). Besides, immune checkpoints correlation analysis displayed that most of the immune checkpoints were significantly up‐regulated in the C1 subtype (Figure 2C,D). To ensure the results were not biased by the analytical algorithm, six other immune infiltration assessment algorithms were employed to confirm the accuracy of the above results, indicating that C1 is a stable “immune subtype” (Figure 2E).

**FIGURE 2:**
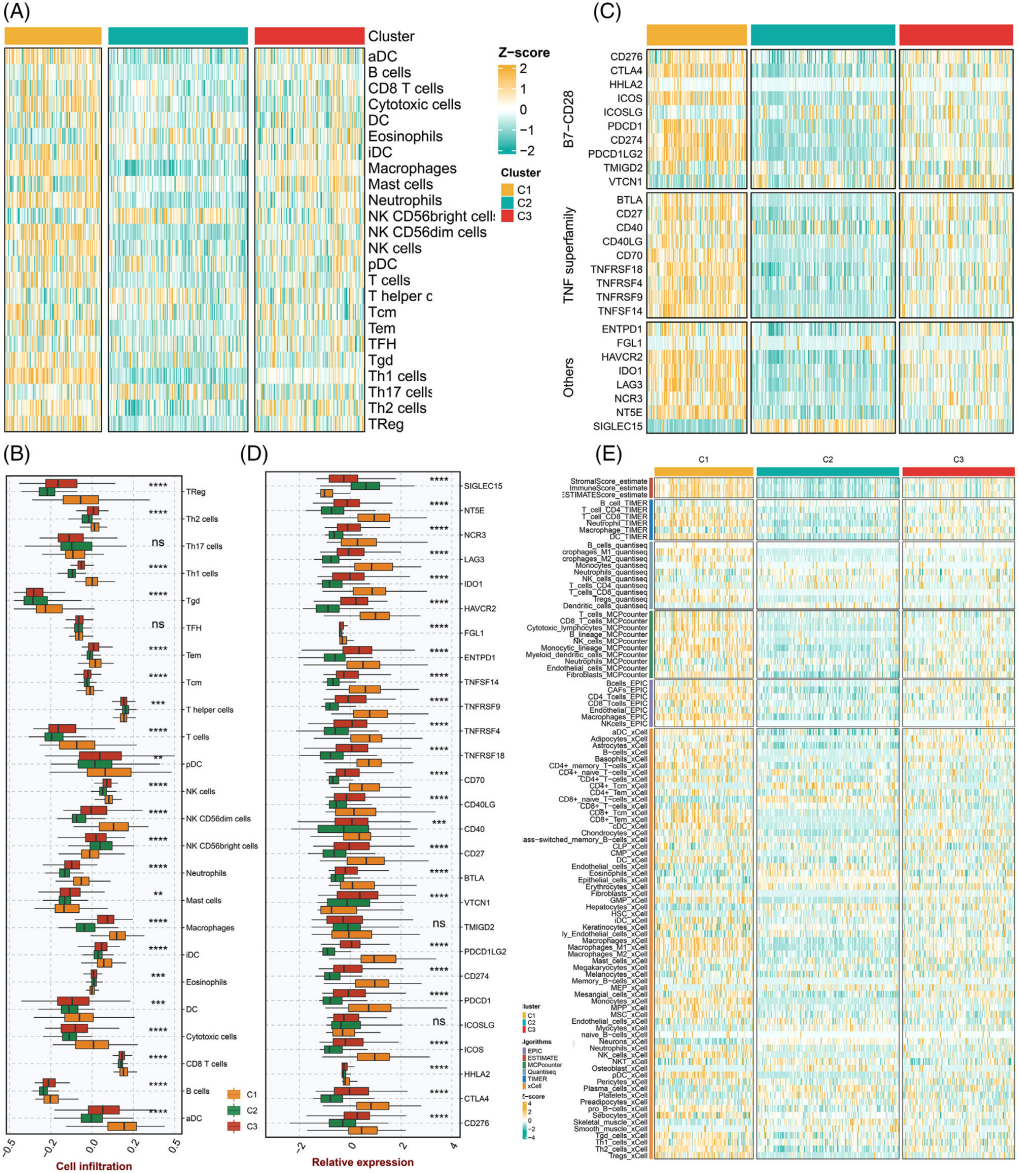
The immune landscape of three BLCA subtypes. (A) Heatmap of 24 immune cells infiltration abundance of BLCA subtypes. (B) Boxplot between BLCA subtypes and 24 immune cells infiltration abundance. (C) Heatmap of 27 immune checkpoint profiles for BLCA subtypes. (D) Boxplot of 27 immune checkpoint profiles for BLCA subtypes. (E) Heatmap of the remaining six immune cell infiltration assessment algorithms for our subtypes

Considering the high infiltration abundance of CD8 T cells in the C1 subtype, patients in C1 tend to exhibit a better immunotherapy response. To verify this, some immune response predictive signatures were retrieved.^5^, ^6^, ^7^ The results revealed that C1 subtype patients performed significantly higher TIS and APS scores and a higher immune response rate than other subtypes (Figure 3A–C). Furthermore, the result of SubMap analysis indicated that patients with subtype C1 exhibited better immune responses across all 14 immunotherapy cohorts (Figure 3D,E), suggesting that the C1 subtype was a robust immune subtype and displayed a better immune response.

**FIGURE 3:**
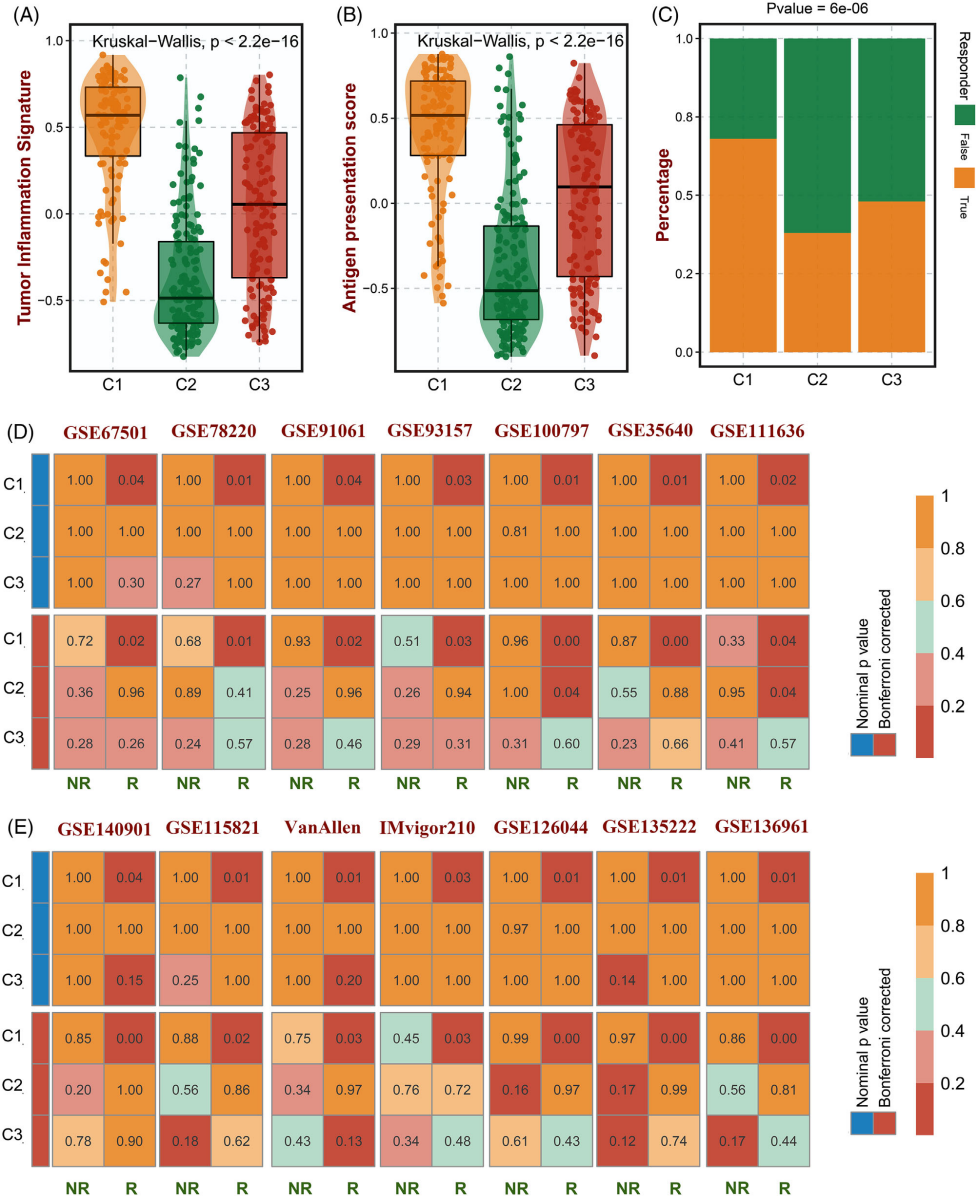
Immunotherapy response prediction and validation. (A–C) The performance of our subtypes in TIS (A), APS (B) and TIDE (C). (D,E) SubMap analysis shows the similarity of gene expression profiles (GEPs) between the BLCA subtypes of the cancer genome atlas (TCGA) and 14 GEO immunotherapy datasets

To characterize genomic alterations in BLCA classifications, we further compared the somatic mutation frequencies and copy number variations (CNVs). As illustrated in Figure S6A–C, C1 exhibited dramatically lower FGA, FGG, and FGL, suggesting that the C1 patients were less regulated by CNVs. Additionally, the characteristic mutations and CNVs of the three subtypes we also investigated (Figure S6D).

To acquire potential therapeutic drugs for specific subtypes of BLCA patients, differential drug‐response analysis was conducted to identify compounds with the lowest area under the curve (AUC) compared with other subtypes. For a detailed description, please refer to Supporting Information. As illustrated in Figure 4A, seven CTRP drugs and 14 PRISM drugs with the lowest estimated AUC values for the C1 subtype were obtained. In parallel, three CTRP compounds and four PRISM compounds with the lowest estimated AUC values for the C2 subtype were finally screened (Figure 4B).

**FIGURE 4:**
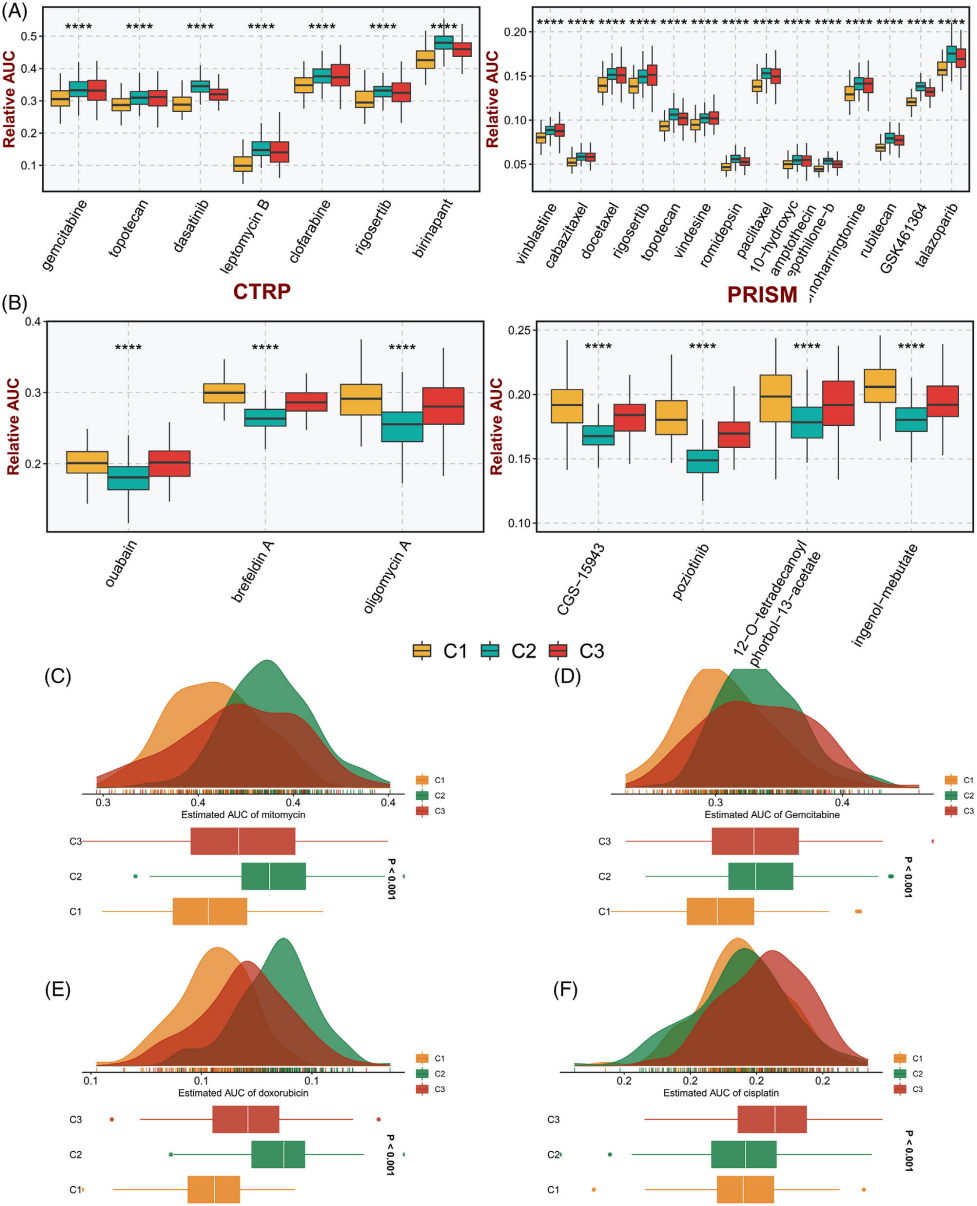
Potential therapeutic agents for specific subtypes. (A,B) Potential therapeutic compounds for C1(A) and C2 (B) BLCA subtypes in CTRP and PRISM databases, respectively. (C–F) the area under the curve (AUC) values of mitomycin (C), gemcitabine (D), doxorubicin (E), and cisplatin (F) in different subtypes

Mitomycin, gemcitabine, and doxorubicin are the most commonly utilized drugs for intravesical chemotherapy, and cisplatin is the standard treatment for systemic chemotherapy in BLCA. The AUC values of mitomycin, gemcitabine, doxorubicin and cisplatin in the C1 subtype were significantly lower, indicating that the C1 subtype exhibited the highest potential benefit from chemotherapy (Figure 4C–F).

Taken together, our classifications were well characterized and performed markedly different clinical and molecular features: (i) C1 subtype: high immune infiltration, revealing better immunotherapy responses, and a higher potential benefit for intravesical and systemic chemotherapy. (ii) C2 subtype: metabolism‐related, lower tumour stage, and good prognosis. (iii) C3 subtype: Wnt pathway‐related, higher tumour stage, poor prognosis, and frequent *HMCN1* mutations.

In conclusion, according to the epigenetically regulated GEPs, we developed three robust molecular subtypes, which not only provided new insights into the close link between BLCA heterogeneity and epigenetics but also provided a promising platform to optimize decision‐making and surveillance protocol for individual BLCA patients.

## CONFLICT OF INTEREST

The authors declare that they have no conflict of interest.

## Supporting information


**Figure S1 The flowchart of this study**. Three well‐characterized BLCA subtypes were identified and validated in five independent cohorts by integrative clustering of MIRcor and METcor gene expression profiles (GEPs). These BLCA subtypes exhibit significantly different clinical and molecular features. Afterwards, the correlation between our classifications and clinical features, published subtypes, epigenetic and genomic features, immune landscape, immunotherapy response, and subtype‐specific potential therapeutic agents were further investigated.
**Figure S2 Identification of METcor and MIRcor genes in BLCA**. (**A**) Overlap of the METcor and MIRcor genes. (**B,C**) The proportional frequencies of promoter CpG sites based on their distance relative to CpG islands (**B**) and genomic locations (**C**). Shore, 0–2 kb upstream or downstream from CpG island; Shelf, 2–4 kbp upstream or downstream from CpG island; Opensea, other regions of the genome. (**D,E**) Pathway analyses of the METcor (**D**) and MIRcor (**E**) genes, respectively. (**F**) Correlation between the frequencies of aberrant METcor and MIRcor genes in each sample of the TCGA dataset. (**G**) Pairwise correlations among the frequencies of METcor_high, METcor_low, MIRcor_high and MIRcor_low genes, respectively.
**Figure S3 Aberrant gene frequencies in different subtypes**. (**A**) All METcor and MIRcor genes; (**B**) METcor genes; and (**C**) MIRcor genes.
**Figure S4. Functional and clinical characteristics of the BLCA subtypes in the TCGA and GEO datasets**. (**A‐D**) Potential functional and molecular characteristics of different subtypes. (**E,F**) Correlations of our subtypes with clinical characteristics and previous BLCA classifications in the TCGA‐BLCA (**E**) and GSE13507 (**F**) datasets.
**Figure S5. Validation of the functional characteristics for each subtype via GSEA‐based GO and KEGG analysis. (A‐B)** Results of GO(**A**) and KEGG (**B**) enrichment analysis for C1 subtype. **(C,D)** Results of GO (**C**) and KEGG (**D**) enrichment analysis for C2 subtype. **(E,F)** Results of GO (**E**) and KEGG (**F**) enrichment analysis for C3 subtype. GSEA: Gene Set Enrichment Analysis; GO: Gene Ontology; KEGG: Kyoto Encyclopedia of Genes and Gnomes.
**Figure S6. Somatic mutational and CNVs landscape with regard to BLCA subtypes**. (**A–C**) The fraction of genomic alterations (FGA) (**A**), genomes gained (FGG) (**B**), and genomes lost (FGL) (**C**) in distinct BLCA subtypes. (**D**) The mutational landscape of the top 20 frequently mutated genes and the CNV landscape of the top 20 AMP and Holmdel chromosome fragments for different classifications.

Supporting Information
