# Supplementary material for: Epigenetically regulated gene expression profiles recognized three molecular classifications with prognostic and therapeutic implications in bladder cancer
Source: Clin Transl Med. 2023 Mar 2;13(3):e1145. doi: 10.1002/ctm2.1145 (PMC9982075; doi:10.1002/ctm2.1145)
Supplement: Supplementary file 1 — Figure S1 The flowchart of this study. Three well‐characterized BLCA subtypes were identified and validated in five independent cohorts by integrative clustering of MIRcor and METcor gene expression profiles (GEPs). These BLCA subtypes exhibit significantly different clinical and molecular features. Afterwards, the correlation between our classifications and clinical features, published subtypes, epigenetic and genomic features, immune landscape, immunotherapy response, and subtype‐specific potential therapeutic agents were further investigated. Figure S2 Identification of METcor and MIRcor genes in BLCA. (A) Overlap of the METcor and MIRcor genes. (B,C) The proportional frequencies of promoter CpG sites based on their distance relative to CpG islands (B) and genomic locations (C). Shore, 0–2 kb upstream or downstream from CpG island; Shelf, 2–4 kbp upstream or downstream from CpG island; Opensea, other regions of the genome. (D,E) Pathway analyses of the METcor (D) and MIRcor (E) genes, respectively. (F) Correlation between the frequencies of aberrant METcor and MIRcor genes in each sample of the TCGA dataset. (G) Pairwise correlations among the frequencies of METcor_high, METcor_low, MIRcor_high and MIRcor_low genes, respectively. Figure S3 Aberrant gene frequencies in different subtypes. (A) All METcor and MIRcor genes; (B) METcor genes; and (C) MIRcor genes. Figure S4. Functional and clinical characteristics of the BLCA subtypes in the TCGA and GEO datasets. (A‐D) Potential functional and molecular characteristics of different subtypes. (E,F) Correlations of our subtypes with clinical characteristics and previous BLCA classifications in the TCGA‐BLCA (E) and GSE13507 (F) datasets. Figure S5. Validation of the functional characteristics for each subtype via GSEA‐based GO and KEGG analysis. (A‐B) Results of GO(A) and KEGG (B) enrichment analysis for C1 subtype. (C,D) Results of GO (C) and KEGG (D) enrichment analysis for C2 subtype. (E,F) Results of GO (E) and KEG [file CTM2-13-e1145-s002.docx]

**Supplementary Figures**

- **Figure S1**
- **Figure S2**
- **Figure S3**
- **Figure S4**
- **Figure S5**
- **Figure S6**


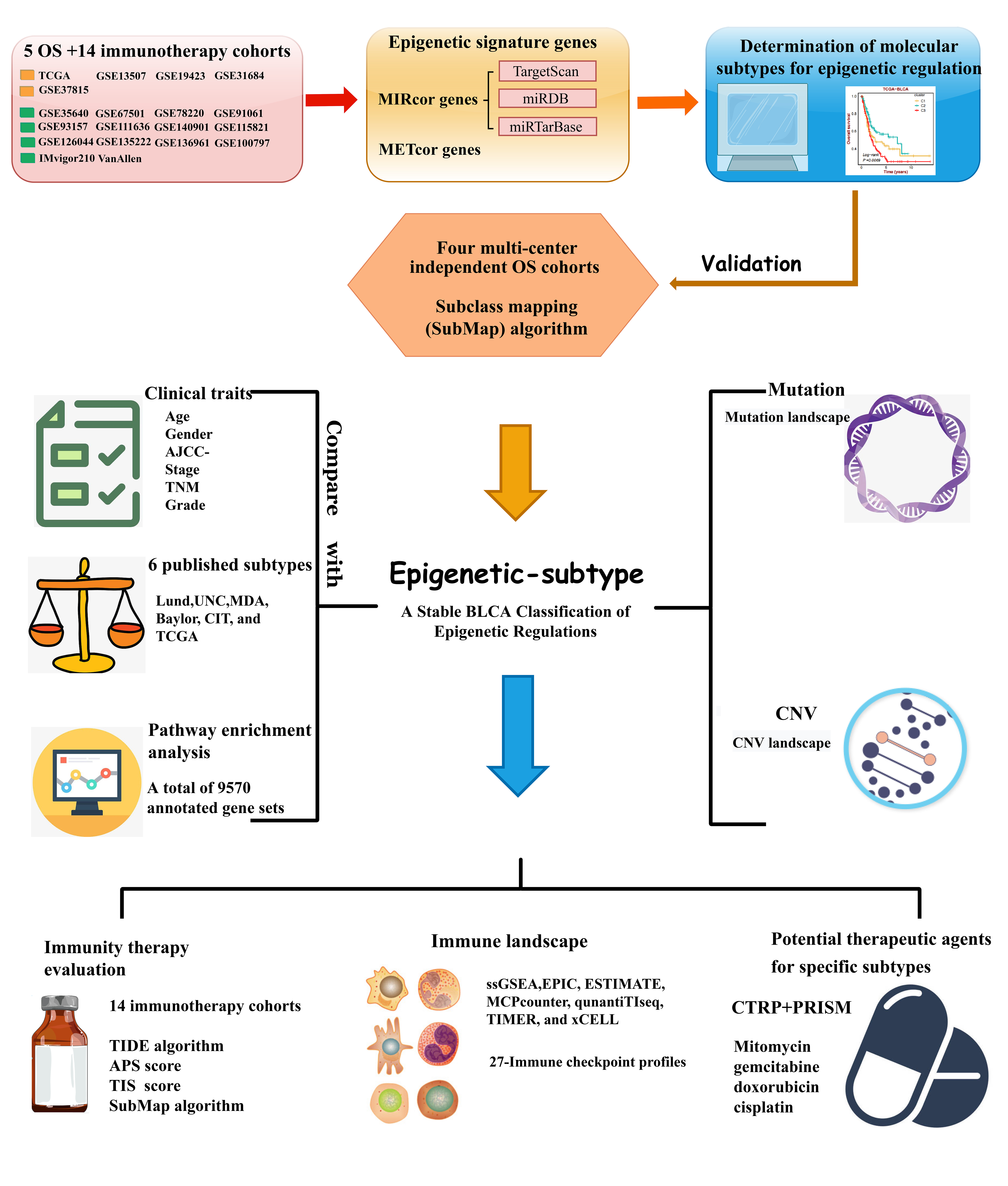


**Figure S1. The flowchart of this study.** Three well-characterized BLCA subtypes were identified and validated in five independent cohorts by integrative clustering of MIRcor and METcor gene expression profiles (GEPs). These BLCA subtypes exhibit significantly different clinical and molecular features. Afterward, the correlation between our classifications and clinical features, published subtypes, epigenetic and genomic features, immune landscape, immunotherapy response, and subtype-specific potential therapeutic agents were further investigated.


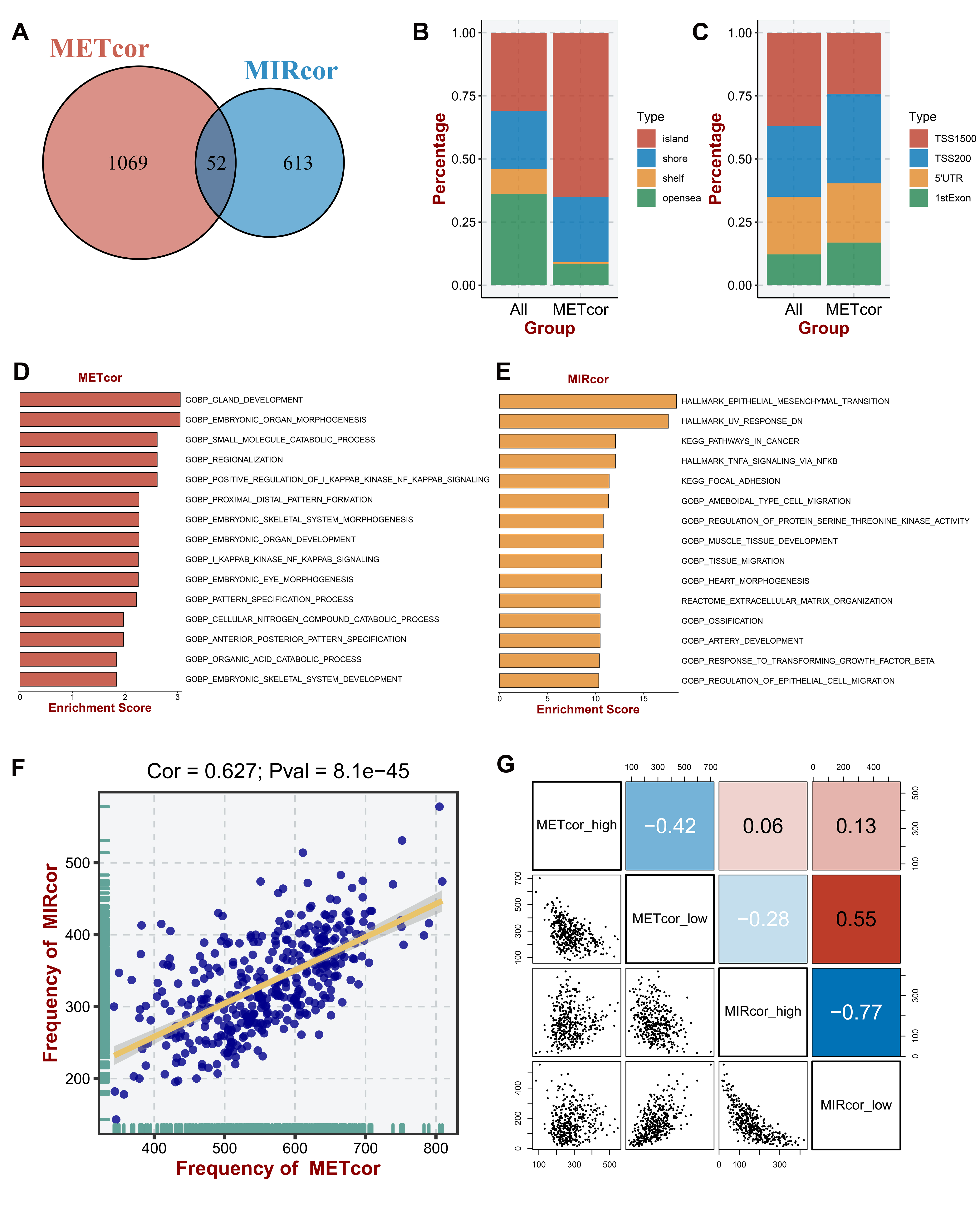


**Figure S2. Identification of METcor and MIRcor genes in BLCA.** (**A**) Overlap of the METcor and MIRcor genes. (**B-C**) The proportional frequencies of promoter CpG sites based on their distance relative to CpG islands (**B**) and genomic locations (**C**). Shore, 0–2 kb upstream or downstream from CpG island; Shelf, 2–4 kbp upstream or downstream from CpG island; Opensea, other regions of the genome. (**D-E**) Pathway analyses of the METcor (**D**) and MIRcor (**E**) genes, respectively. (**F**) Correlation between the frequencies of aberrant METcor and MIRcor genes in each sample of the TCGA dataset. (**G**) Pairwise correlations among the frequencies of METcor_high, METcor_low, MIRcor_high and MIRcor_low genes, respectively.


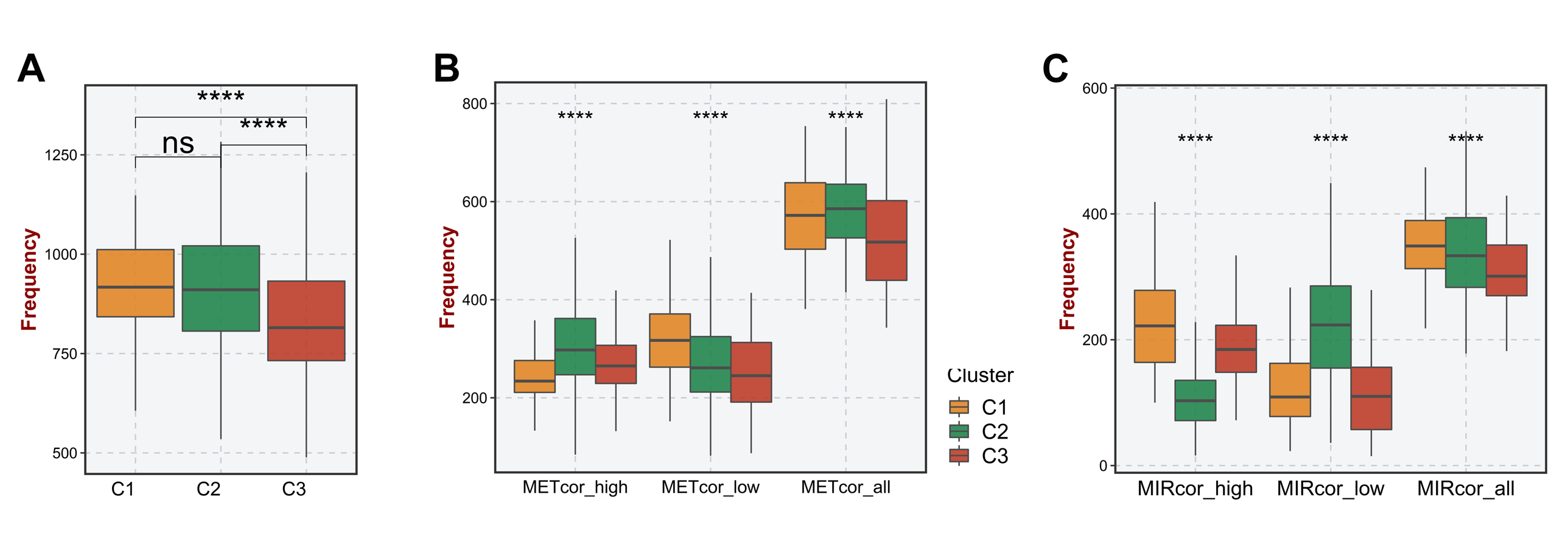


**Figure S3. Aberrant gene frequencies in different subtypes.** (**A**) All METcor and MIRcor genes; (**B**) METcor genes; and (**C**) MIRcor genes.


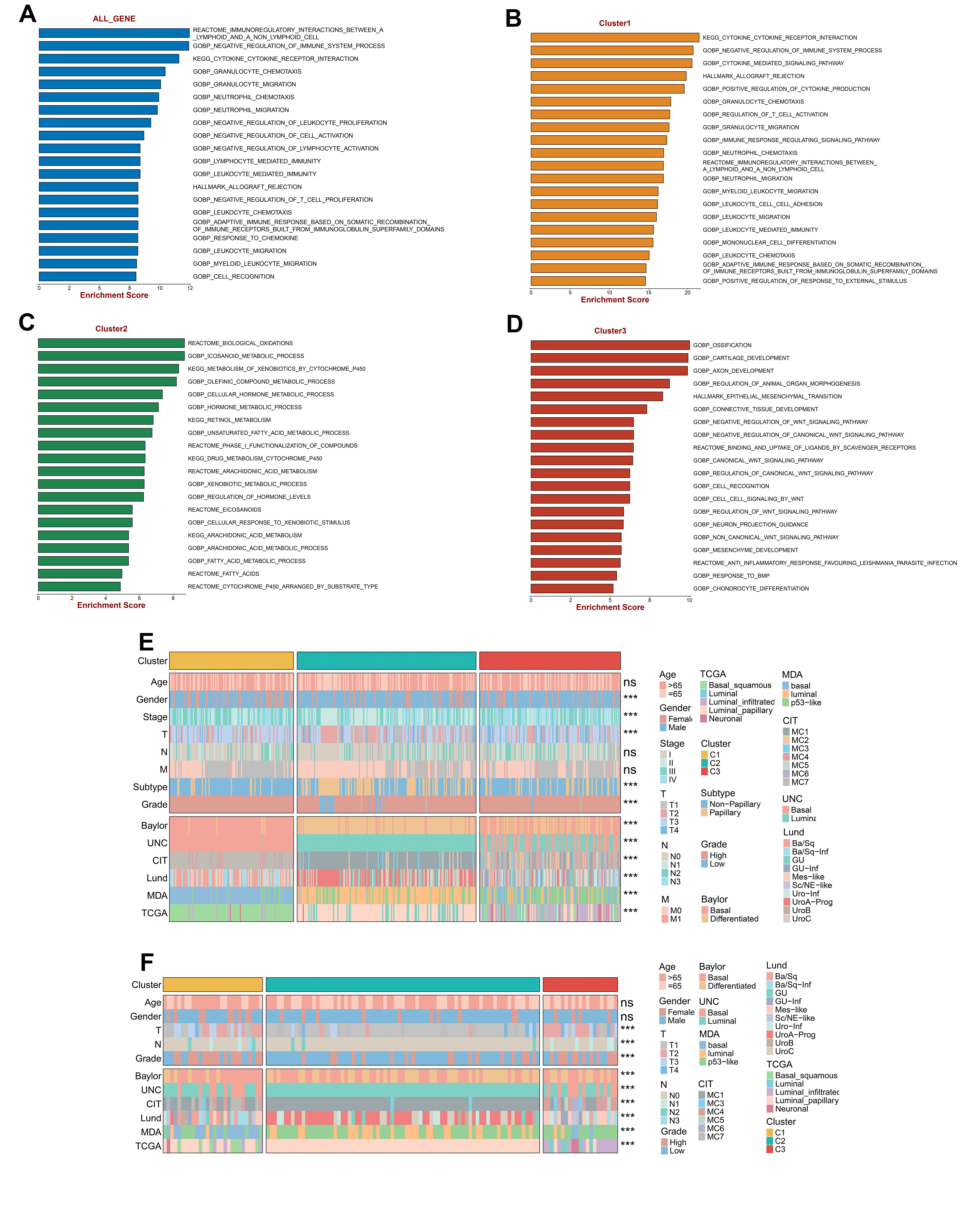


**Figure S4. Functional and clinical characteristics of the BLCA subtypes in the TCGA and GEO datasets.** (**A-D**) Potential functional and molecular characteristics of different subtypes. (**E-F**) Correlations of our subtypes with clinical characteristics and previous BLCA classifications in the TCGA-BLCA (**E**) and GSE13507 (**F**) datasets.





**Figure S5. Validation of the functional characteristics for each subtype via GSEA-based GO and KEGG analysis. (A-B)** Results of GO(**A**) and KEGG(**B**) enrichment analysis for C1 subtype. **(C-D)** Results of GO(**C**) and KEGG(**D**) enrichment analysis for C2 subtype. **(E-F)** Results of GO(**E**) and KEGG(**F**) enrichment analysis for C3 subtype. GSEA: Gene Set Enrichment Analysis; GO: Gene Ontology; KEGG: Kyoto Encyclopedia of Genes and Gnomes.


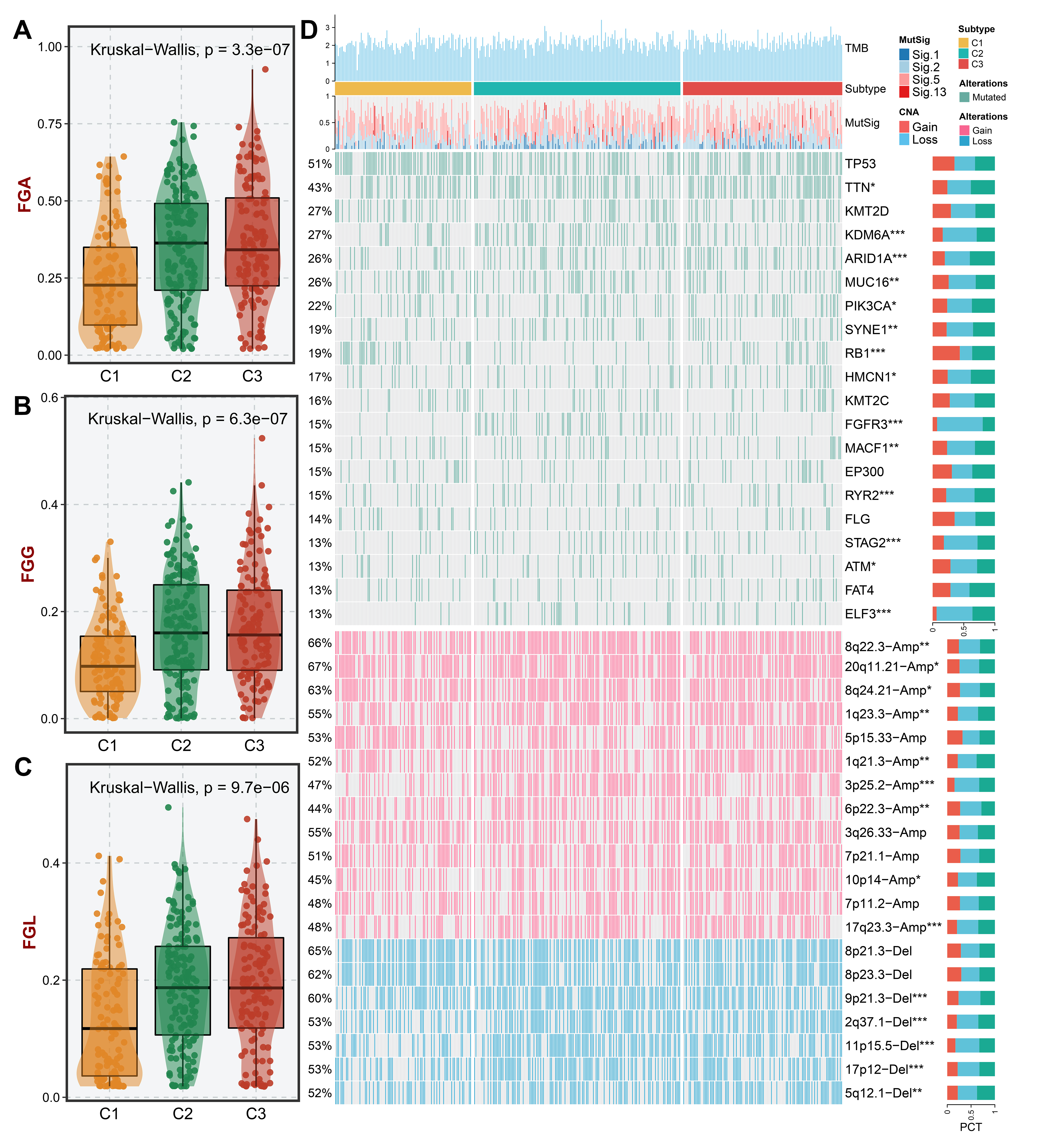


**Figure S6. Somatic mutational and CNVs landscape with regard to BLCA subtypes.** (**A-C**) The fraction of genomic alterations (FGA) (**A**), genomes gained (FGG) (**B**), and genomes lost (FGL) (**C**) in distinct BLCA subtypes. (**D**) The mutational landscape of the top 20 frequently mutated genes and the CNV landscape of the top 20 AMP and Homdel chromosome fragments for different classifications.
