# Supplementary material for: Epigenetically regulated gene expression profiles recognized three molecular classifications with prognostic and therapeutic implications in bladder cancer
Source: Clin Transl Med. 2023 Mar 2;13(3):e1145. doi: 10.1002/ctm2.1145 (PMC9982075; doi:10.1002/ctm2.1145)
Supplement: Supplementary file 2 — Supporting Information [file CTM2-13-e1145-s001.docx]

**Materials and methods**

**Data collection and processing**

The flowchart of our research is illustrated in Figure S1. A total of five independent datasets containing overall survival information (n=721, including GSE31684, GSE19423, GSE37815, GSE13507 and TCGA-BLCA) were retrospectively retrieved from the Gene Expression Omnibus (GEO) and The Cancer Genome Atlas (TCGA) database, respectively. The HumanMethylation450 array, miRNA expression and somatic mutation were collected from the TCGA GDC database. Copy number variations (CNVs) data processed by the Genomic Identification of Significant Targets in Cancer 2.0 (GISTIC2.0) algorithm was retrieved from FireBrowse (http://firebrowse.org/)^1^. Of note, the FPKM normalized data from TCGA were further converted into log2 (TPM + 1). To assess the predictive performance of subtypes on immune response, a total of 14 cohorts (n=822) containing immunotherapy information, including GSE35640, GSE67501, GSE78220, GSE91061, GSE93157, SGE100797, GSE111636, GSE115821, GSE126044, GSE135222, GSE136691, GSE140901, IMvigor210, and VanAllen cohorts were comprehensively retrieved.

**Identification of METcor and MIRcor genes**

The METcor and MIRcor genes were identified as following steps:

(1). Based on 396,065 methylated-CpG sites and 15,996 mRNAs of the TCGA-BLCA cohort, 85,595 pairs of interacting CpG sites and mRNAs (n=11545) located in the promoter region were screened. Meanwhile, 21265,143 pairs of interacting miRNAs (n = 6061) and mRNAs (n = 19350) in the TargetScan database, 938,564 pairs of interacting miRNAs (n = 2586) and mRNAs (n = 17390) in the miRDB database, and 322,135 pairs of interacting miRNAs (n = 2597) and mRNAs (n = 14886) in the miRTarBase database were retrieved, respectively. Afterward, 44,033 intersection miRNA-mRNA pairs over these three databases were obtained. A total of 35,823 pairs of interacting miRNAs (n = 1882) and mRNAs (n = 6418) were further acquired after taking the intersection with the TCGA cohort.

(2). Next, the Pearson correlation coefficients for miRNA-mRNA and CpG-mRNA pairs were calculated separately. To stabilize variance, Fisher Z-transformation was employed to normalize the correlation coefficient.

(3). As described in the study by Wang et al.^2^, although the overall negative correlation was only observed in CpG-miRNA pairs (z-test, P < 0.001), not miRNA-mRNA pairs (z-test, P = 0.604). We only retained negatively correlated miRNAs-mRNA pairs for the following reasons: (i) It is well known that miRNAs tend to exert biological functions by inhibiting the expression of mRNA^3^. (ii) The complex mechanisms underlying the positive correlation of miRNA-mRNA interactions have not been extensively validated in in vivo and in vitro experiments^4^. (iii) The positive correlation of miRNA-mRNA is probably produced by other undiscovered factors. For instance, the results of H et al. suggested that only a small fraction of miRNA-mRNA positive correlations observed in patients were validated in wet experiments^5^, indicating that there may be additional interfering factors in the positive miRNA-mRNA correlations. Finally, MIRcor and METcor genes were identified based on Fisher Z-transformed correlation coefficients with 95% confidence intervals (P < 0.05, < -1.96).

Additionally, the relationship between the MIRcor and METcor genes was further investigated. Patients were ranked from low to high based on each MIRcor or METcor gene expression. The gene was defined as low-expression gene in the top quartile of patients, whereas high-expression gene in the bottom quartile. As illustrated in Figure S2F, frequently abnormal MIRcor gene patients tend to exhibit frequent aberration of METcor gene. Besides, pairwise comparisons revealed that this positive correlation is mainly concentrated in METcor and MIRcor low expression genes aberrant frequencies (Figure S2G), suggesting that the aberrant frequencies of MIRcor genes and METcor genes were significantly correlated in BLCA.

**Determination of molecular subtypes for epigenetic regulation**

Based on multi-omics data from METcor and MIRcor genes, iClusterBayes, an integrative clustering method implemented by the *iClusterPlus* R package was employed to recognize BLCA subtypes. The optimal number of classifications was identified via the deviance ratio plots and the Bayesian information criteria (BIC).

**Screening of signature genes for BLCA classification**

To determine the signature genes for each subtype, differential gene expression analysis among distinct subtypes via a linear model implemented in the *limma* package was further performed. Genes were arranged in descending order based on the value of logFC, respectively. To solve the problem of over-fitting caused by too many signature genes and under-fitting caused by too few signature genes, the top 500 differential expressed genes (all P< 0.05) were defined as characteristic genes for each subtype separately.

**Validation of molecular subtypes based on multi-center independent cohorts**

Based on the eigengenes screened in the TCGA cohort, we employed the nearest template prediction (NTP) algorithm to classify the GEO cohort with prognostic information^6^. Meanwhile, subclass mapping (SubMap) algorithm, a method to evaluate the similarity of subtypes between different cohorts based on GEPs was utilized to further validate whether the subtypes obtained in GEO and TCGA cohorts were consistent.

**Pathway enrichment analysis**

To investigate the potential biological mechanism of subtypes, pathway enrichment analysis was conducted via *clusterProfiler* package. A total of 9570 annotated gene sets were retrieved from the Molecular Signatures Database (MSigDB), including 50 cancer hallmark gene sets, 186 KEGG gene sets, 196 PID gene sets, 289 Biocarta gene sets, 1499 Reactome gene sets, and 7350 GO biological process gene sets. In addition, to further verify the biological properties of each subtype, the GSEA algorithm was implemented to analysis the enrichment of different subtypes in GO and KEGG.

**Associations of subtypes with clinical traits, published subtypes, miRNA expression, and DNA methylation**

Next, we explored the correlation of molecular subtypes with common clinical features such as age, sex, stage, T, N, M, grade, etc. In addition, *BLCAsubtyping*, a transcriptomic R package to stratify BLCA patients based on six published molecular subtypes (Lund, UNC, MDA, Baylor, CIT, and TCGA), was utilized to investigate the correlation of our subtype with 6 published subtypes^7^. To investigate miRNA expression and promoter DNA methylation patterns in BLCA subtypes, the average levels of miRNA expression and promoter CpG methylation between each subtype and normal samples were further analyzed.

**The immune landscape of molecular subtypes**

The single-sample gene set enrichment analysis (ssGSEA) algorithm was employed to assess the infiltration abundance of 24 immune cells in tumor immune microenvironment via *GSVA* package in the TCGA-BLCA cohort. Gene set of 24-immune cell types and 27-immune checkpoints, including the member of the TNF superfamily, B7-CD28 family, and other molecules, were retrieved from the published research^8, 9, 10, 11, 12^. Afterward, the relationship between the molecular subtypes and immune infiltration and checkpoints was investigated. Of note, to ensure the analytical algorithm did not bias the results, six other immune infiltration assessment algorithms, including EPIC, ESTIMATE, MCPcounter, qunantiTIseq, TIMER, and xCELL were employed to confirm the accuracy of ssGSEA results.

**Immunotherapy response prediction and validation**

To assess the relationship between molecular subtypes and immune response, several immune response predictive signatures were systematically collected, including the tumor immune dysfunction and exclusion (TIDE), antigen presentation score (APS), and tumor inflammation signature (TIS)^13, 14, 15^. Besides, SubMap analysis was utilized to assess the similarity for gene expression profiles(GEPs) in the identified BLCA classifications and 14 immunotherapy cohorts, thereby validating the immune responses of the different subtypes.

**The multi-omics landscape of subtypes based on somatic mutation and CNVs**

To investigate the differences in genomic mutations with regard to different subtypes, the mutation waterfall plot of the top 20 genes with the highest mutation number in BLCA was visualized via the *maftools* and *ComplexHeatmap* R packages. Meanwhile, the *deconstructSigs* package was performed to infer the mutation signature in subtypes^16^. For CNVs, the ComplexHeatmap package was performed to visualize the CNV waterfall chart of the top 20 amplification (AMP) and homozygous deletion (Homdel) chromosome fragments. To investigate the proportion of genomic alterations, the fraction of genomic alterations (FGA), genomes gained (FGG), and genomes lost (FGL) were also calculated.

**Estimation of drug response and potential therapeutic agents**

Drug sensitivity data of cancer cell lines were collected from PRISM ([https://depmap.org/po rtal/prism/](https://depmap.org/po%20rtal/prism/)) and the cancer therapeutics response portal datasets (CTRP, [https://portals.broadinstitute.org/ctrp/?page=#ctd2BodyHome](https://portals.broadinstitute.org/ctrp/?page=#ctd2BodyHome)). The CTRP contains the sensitivity data for 481 compounds over 835 CCLs and the PRISM contains the sensitivity data for 1448 compounds over 482 CCLs. Both two datasets provide the area under the dose-response curve (area under the curve-AUC) values as a measure of drug sensitivity, and lower AUC values indicate increased sensitivity to treatment. After removing compounds with more than 20% of samples with missing values and cell lines from hematopoietic and lymphoid tissues, 266 and 1285 compounds were obtained in the CTRP and PRISM databases, respectively. Afterward, based on the expression profile, the *pRRophetic* package based on the ridge regression algorism was employed to predict the drug response, resulting in an estimated AUC value for each compound in the sample. To ensure that the obtained drug sensitivities were reliable, cisplatin, a commonly applied drug in neoadjuvant chemotherapy for BLCA was utilized to explore whether the predicted drug sensitivity was consistent with its clinical efficacy. Studies have shown that reduced expression or dysfunction of *BRCA1* predicts a higher cisplatin sensitivity in BLCA^17^. Thus, patients in the TCGA cohort were divided into high- and low-*BRCA* expression groups based on the median values of *BRCA1* expression. In line with the published research, low *BRCA1* expression patients exhibited a lower cisplatin AUC (P<0.001) (the figure below), indicating the remarkable accuracy of estimated drug response.

**
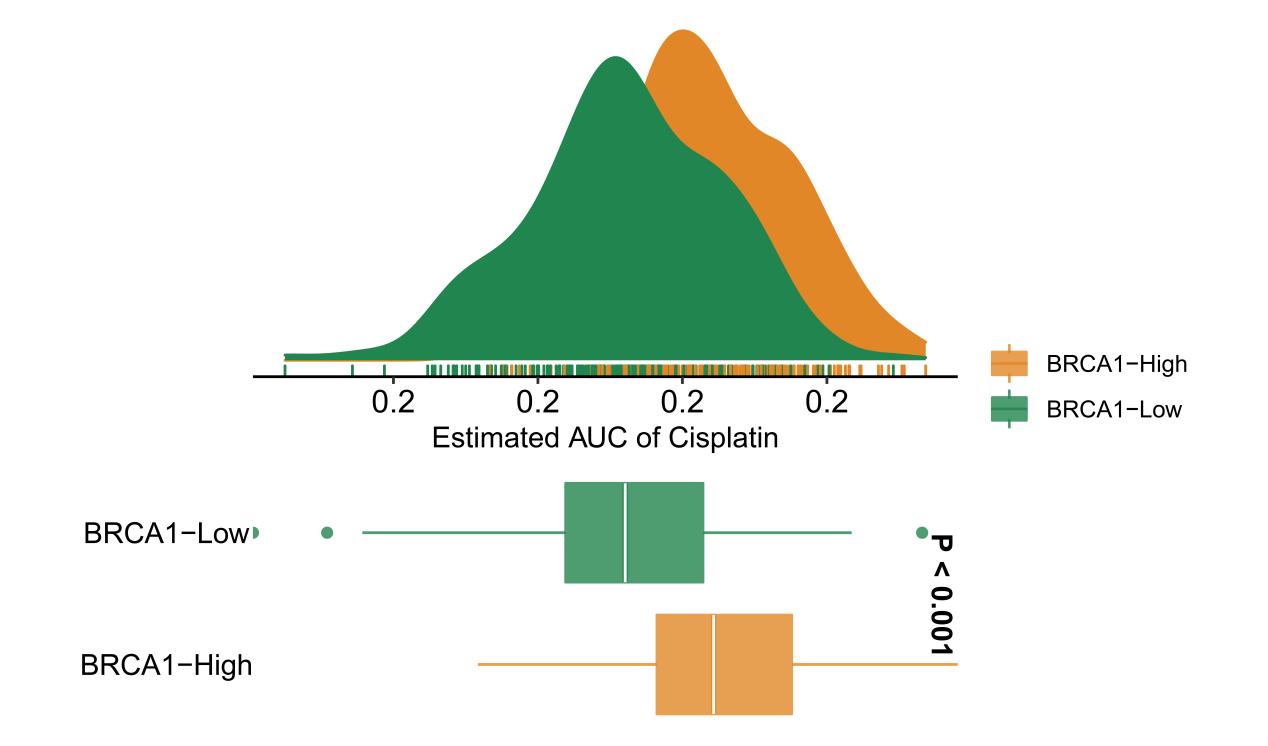
**

**Comparison of estimated cisplatin’s sensitivity between high and low *BRCA1* expression groups.**

**Statistical analysis**

The relationship between two variables was calculated by Pearson correlation. The *Survival* R package was utilized to perform Kaplan-Meier survival analysis and the different significance was determined by the log-rank test. Differences in subtypes were compared by Kruskal–Walli’s rank sum test. P <0.05 was regarded as statistically significant. All statistical P values were two-sided and adjusted P-value was employed using Benjamini-Hochberg (BH) multiple tests. All data processing and plotting were finished in R 4.1.2 software.

**Reference**

1. Mermel CH, Schumacher SE, Hill B, Meyerson ML, Beroukhim R, Getz G. GISTIC2.0 facilitates sensitive and confident localization of the targets of focal somatic copy-number alteration in human cancers. *Genome Biol*. 2011; 12(4):R41.

2. Wang X, Liu J, Wang D, Feng M, Wu X. Epigenetically regulated gene expression profiles reveal four molecular subtypes with prognostic and therapeutic implications in colorectal cancer. *Brief Bioinform*. 2021; 22(4).

3. Matsuyama H, Suzuki HI. Systems and Synthetic microRNA Biology: From Biogenesis to Disease Pathogenesis. *Int J Mol Sci*. 2019; 21(1).

4. Tan H, Kim P, Sun P, Zhou X. miRactDB characterizes miRNA-gene relation switch between normal and cancer tissues across pan-cancer. *Brief Bioinform*. 2021; 22(3).

5. Tan H, Huang S, Zhang Z, Qian X, Sun P, Zhou X. Pan-cancer analysis on microRNA-associated gene activation. *EBioMedicine*. 2019; 43:82-97.

6. Hoshida Y. Nearest template prediction: a single-sample-based flexible class prediction with confidence assessment. *PLoS One*. 2010; 5(11):e15543.

7. Kamoun A, de Reyniès A, Allory Y, et al. A Consensus Molecular Classification of Muscle-invasive Bladder Cancer. *Eur Urol*. 2020; 77(4):420-433.

8. Chrétien S, Zerdes I, Bergh J, Matikas A, Foukakis T. Beyond PD-1/PD-L1 Inhibition: What the Future Holds for Breast Cancer Immunotherapy. *Cancers (Basel)*. 2019; 11(5).

9. Wang J, Sanmamed MF, Datar I, et al. Fibrinogen-like Protein 1 Is a Major Immune Inhibitory Ligand of LAG-3. *Cell*. 2019; 176(1-2).

10. Janakiram M, Chinai JM, Zhao A, Sparano JA, Zang X. HHLA2 and TMIGD2: new immunotherapeutic targets of the B7 and CD28 families. *Oncoimmunology*. 2015; 4(8):e1026534.

11. Bindea G, Mlecnik B, Tosolini M, et al. Spatiotemporal dynamics of intratumoral immune cells reveal the immune landscape in human cancer. *Immunity*. 2013; 39(4):782-795.

12. Ward-Kavanagh LK, Lin WW, Šedý JR, Ware CF. The TNF Receptor Superfamily in Co-stimulating and Co-inhibitory Responses. *Immunity*. 2016; 44(5):1005-1019.

13. Thorsson V, Gibbs DL, Brown SD, et al. The Immune Landscape of Cancer. *Immunity*. 2018; 48(4).

14. Charoentong P, Finotello F, Angelova M, et al. Pan-cancer Immunogenomic Analyses Reveal Genotype-Immunophenotype Relationships and Predictors of Response to Checkpoint Blockade. *Cell Rep*. 2017; 18(1):248-262.

15. Jiang P, Gu S, Pan D, et al. Signatures of T cell dysfunction and exclusion predict cancer immunotherapy response. *Nat Med*. 2018; 24(10):1550-1558.

16. Alexandrov LB, Nik-Zainal S, Wedge DC, et al. Signatures of mutational processes in human cancer. *Nature*. 2013; 500(7463):415-421.

17. Font A, Taron M, Gago JL, et al. BRCA1 mRNA expression and outcome to neoadjuvant cisplatin-based chemotherapy in bladder cancer. *Annals of oncology : official journal of the European Society for Medical Oncology*. 2011; 22(1):139-144.
